# Variable phenotypic presentations of renal involvement in Fabry disease: a case series

**DOI:** 10.12688/f1000research.13708.1

**Published:** 2018-03-22

**Authors:** Sarah McCloskey, Paul Brennan, John A Sayer

**Affiliations:** 1 Renal Services, Newcastle Upon Tyne Hospitals NHS Trust, Newcastle upon Tyne, Tyne and Wear , NE7 7DN, UK; 2 Northern Genetics Service, Central Parkway, Newcastle, NE1 3BZ, UK; 3 Institute of Genetic Medicine, Newcastle University, Newcastle upon Tyne, Tyne and Wear, NE1 3BZ, UK

**Keywords:** Fabry, chronic kidney disease, proteinuria, parapelvic cysts, GLA, mutation

## Abstract

Fabry disease is an X-linked genetic deficiency in the alpha-galactosidase enzyme resulting in intracellular accumulation of glycosphingolipids and multisystem organ dysfunction. Typically 50% of males and 20% of affected females have renal involvement, ranging from proteinuria or reduced renal function, renal parapelvic cysts and progressive renal disease ultimately requiring transplantation or dialysis. The phenotypic presentation of Fabry disease is incredibly varied and will even vary between family members with the same confirmed genetic mutation. In a cohort of patients affected by Fabry disease in the North East of England we examine the different phenotypic presentations of eight index cases (6 male, 2 female) with predominantly renal disease and the renal manifestations within their family members. The mean age of presentation was 40 years of age (range 23-59 years). Various multisystem manifestations were observed including cardiac, neurological, cerebrovascular and skin involvement. Two of the male index patients reached end stage renal disease (ESRD) requiring renal replacement therapy. Two female index patients had phenotypes limited to hypertension and proteinuria at presentation and the remaining patients had either stable or progressive chronic kidney disease at the time of diagnosis. We demonstrate the need for a high index of suspicion in order to consider Fabry disease as a diagnosis and the importance of cascade genetic screening to identify affected family members so that treatment can be initiated in a timely fashion.

## Introduction

Fabry disease (also called Anderson-Fabry disease) is an X-linked genetic deficiency in the alpha-galactosidase enzyme resulting in an intracellular accumulation of globotriaosylceramide (Gb3) and related glycosphingolipids causing organ dysfunction ^1^. Classic manifestations include acroparesthesia (burning/tingling/numbness at extremities), angiokeratoma (typically over the bathing suit area but can be focal or diffuse) and corneal lipid accumulation known as cornea verticillata or Fleischer vortex dystrophy (seen on slit-lamp examination) ^2^. The condition can also result in progressive renal impairment, gastrointestinal symptoms, heart disease and neurological involvement, including early stroke.

Symptoms of Fabry disease can present in early childhood with overt organ dysfunction usually apparent by the second or third decade, however the phenotype is often variable even within single families ^3^. Furthermore, female heterozygotes often have a degree of alpha-galactosidase activity present resulting in later onset of milder symptoms. Similarly, male patients with atypical variants due to preserved low levels of alpha-galactosidase may present later, typically in the third or fourth decade ^2^.

It is partly this wide variation in clinical presentation that often leads to a delay in diagnosis. Fabry disease remains a rare disease and a high index of clinical suspicion is required to make a new diagnosis. However, with the advent of enzymatic and genetic screening more patients are being identified through a process of cascade screening allowing earlier initiation of treatment. Each index case diagnosed will typically lead to 3–4 cases on cascade screening ^4^. Study data suggests that enzyme replacement therapy (ERT) may slow progression of renal dysfunction if instituted early however it is of less benefit when patients already have significantly reduced renal function and proteinuria ^5– 7^.

As many as 50% males and 20% of females with Fabry disease have renal manifestations ^2^. Proteinuria and reduced estimated glomerular filtration rate (eGFR) may be present in early childhood ^8^. If a renal biopsy is undertaken the pathognomonic histological renal lesion on electron microscopy is that of myelin-like inclusions known as “zebra bodies”. These can be seen in the lysosomes of cells predominantly within the glomerular and distal tubule due to Gb3 deposition. As the kidney disease progresses podocytes and endothelial cells become hypertrophic with foamy appearing vacuoles. Glomerular mesangial thickening then occurs leading to focal and then global glomerulosclerosis which can be detected under light microscopy ^9^. Urinary epithelial cells may also have a vacuolated appearance containing a number of glycosphingolipids droplets and are then known as oval fat bodies.

A number of small studies and population data have demonstrated an increased prevalence of parapelvic and renal sinus cysts in patients with Fabry disease ^10^. The prevalence of cysts increases with age not unlike the prevalence of simple cysts within the general population. The exact aetiology of parapelvic cysts in Fabry disease is unknown. However the specific finding of renal sinus or parapelvic cysts on imaging in a young adult with renal impairment should raise the possibility of the disease as a diagnosis ^11^. Parapelvic cysts resemble renal cortical cysts in morphology but plunge into the renal sinus from the adjacent parenchyma. If large enough they may compress the pelvicalyceal system causing hydronephrosis but due to their hypoechogenicity they may actually be mistaken for hydronephrosis on ultrasound imaging ^12^.

Preferential involvement of the distal tubules relative to other segments results in a reduced ability to concentrate urine, meaning polyuria may be the first manifestation of renal involvement ^13^. However, it is usually the development of proteinuria or renal impairment that initiates referral to renal services / nephrology departments. Proteinuria and renal impairment progress with advancing age and result in end stage renal disease (ESRD) in almost all male patients and a significant proportion of female patients, if untreated. ESRD is the leading cause of death in male patients with untreated Fabry disease ^14^. Heavy proteinuria is more common in adult males and is associated with a more rapid deterioration in renal function ^15^. Although proteinuria is strongly associated with disease progression, it is not always overt in advanced renal disease.

Treatment of Fabry disease consists of providing patients with recombinant human alpha-galactosidase. Two formulations currently exist and appear to be equally efficacious however both have a significant treatment cost. Some clinicians choose to treat classically presenting males (with low or absent alpha-galactosidase levels) as soon as the diagnosis is established in order to prevent disease progression. Due to the limited evidence of benefits of ERT in patients with established renal impairment, the European Best Practice Group do not advocate treatment in patients who already have established renal impairment (eGFR<60mL/min/1.73m ^2^ or proteinuria>1g/day) unless they have non-renal symptoms that require treatment. A new chaperone therapy called migalastat is able to bind and stabilise certain mutant enzymes, allowing their proper trafficking to the lysosomes where they are able to function. Migalastat offers an alternative treatment option where ERT has lost efficacy, for example in cases of antibody formation to ERT. It also has the advantage of being an orally administered drug and has recently been recommended by NICE ( see NICE recommendation for Migalastat). However, the longer-term benefits of this drug are not known. A recent 6 month placebo controlled study of this drug provided disappointing results with no difference in response between drug and placebo ^16^, whilst an 18 month randomised study comparing ERT and open label migalastat produced more encouraging results ^17^.

As with other forms of proteinuric chronic kidney disease (CKD), hypertension should be treated to a target of below 130/80 mmHg. It is reasonable to treat with an Angiotensin-Converting Enzyme inhibitor or Angiotensin Receptor Blockers as anti-proteinuric agents ^18^. Complications of CKD such as anaemia and renal bone disease should be managed in the same way as for other forms of CKD and ultimately patients should be offered dialysis or transplantation if required.

Patients with Fabry disease have an increased mortality on dialysis when compared to other (non-diabetic) causes of ESRD and the condition is associated with a reduced quality of life on dialysis compared to other patient groups ^19^. Transplantation is therefore recommended as first line treatment for patients with ESRD due to Fabry nephropathy. Post transplantation examination of kidney allograft tissue can show Fabry inclusion bodies however they do not appear to cause graft dysfunction ^20^. Dialysis patients with Fabry disease and transplant recipients may still derive benefit from ERT for extra-renal manifestations such as neurological and cardiac disease.

**Figure 1.**
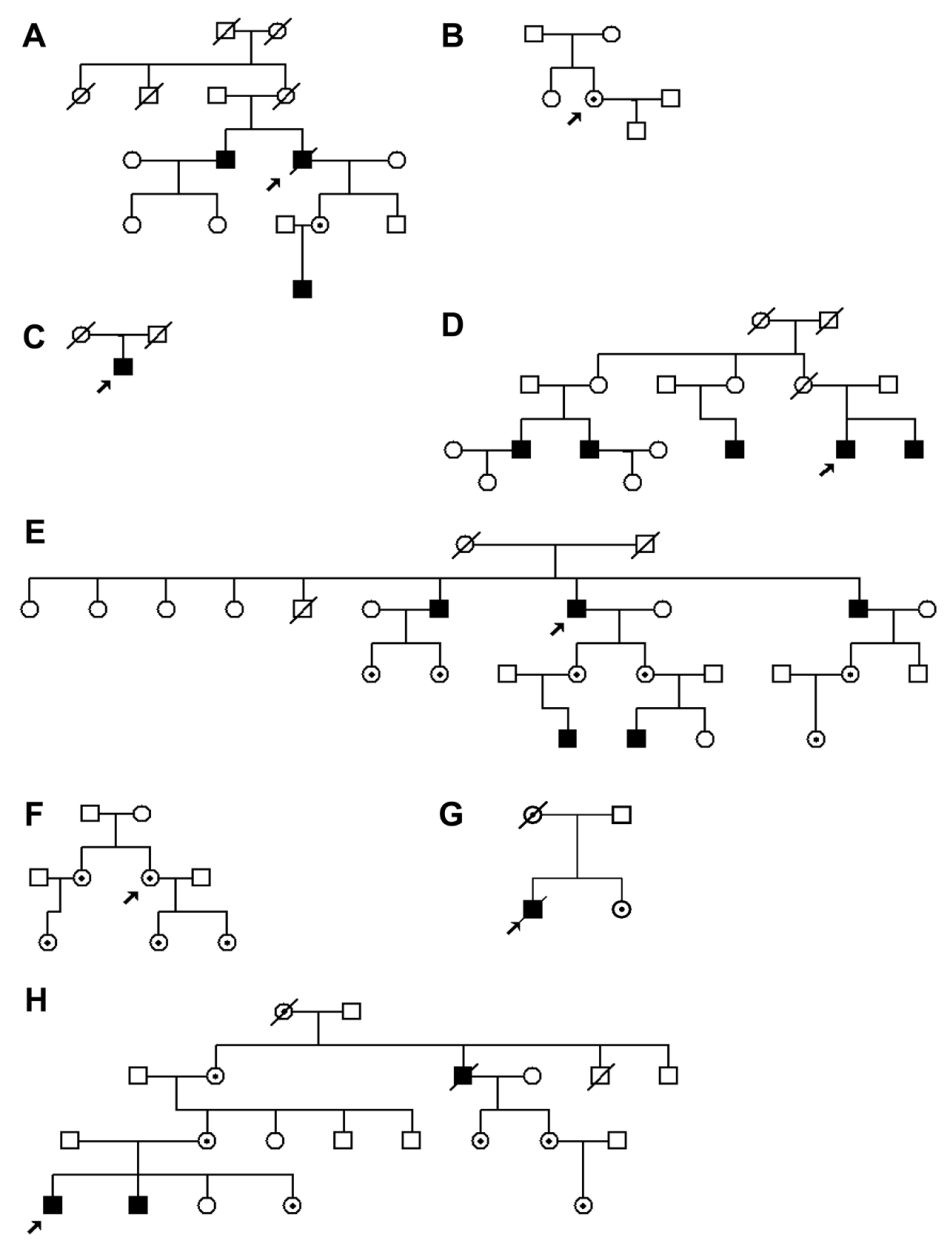
Family pedigree diagrams. Males are represented by squares, females by circles. Affected males are coloured black. Carrier and affected females are marked with a dot. The proband for each family is marked with an arrow.

Within the North East of England there is a sizeable cohort of patients with Fabry disease. Here we review the varying phenotypic presentations of eight patients with overt renal disease and the renal manifestations of Fabry disease within their families ( Table 1). These summaries hopefully help to emphasise the presenting features and patterns of disease progression where the proband has renal features. The importance of a full family history cannot be understated ( Figure 1), as well as looking for evidence of male to male transmission of disease, which would exclude X-linked inherited disease

**Table 1.** Summary of proband presenting features.

Family	Age (years), Sex	Renal	Cardiac	Cerebrovascular	Neurological	Skin	Nucleotide change and rs number if known	Amino Acid change	Reference
A	37, Male	Progressive CKD; ESRD age 40	HCM	-	-	+	c.1235_1236delCT rs797044777	p.Thr412Serfs	*ClinVar: 198402
B	39, Female	Hypertension, proteinuria	-	-	-	-	c.999-1G>C	Splice acceptor mutation	21
C	38, Male	CKD, parapelvic cysts, ESRD aged 48	+	-	-	-	c.547G>A	p.Gly183Ser, splice donor mutation	22
D	50, Male	CKD	HCM	Ischaemic stroke	+		c.350T>G rs12392549	p.Ile117Ser	Novel
E	59, Male	Hypertension, albuminuria	LVH	-	+	-	c.902G>A rs104894828	p.Arg301Gln	23
F	45, Female	CKD, proteinuria	HCM	-	-	-	c.679C>T rs104894841	p.Arg227 *	24
G	23, Male	CKD	LVH	-	-	+	c.334C>T rs104894834	p.Arg112Cys	25
H	32, Male	CKD	-	-	+	+	c.724A>T rs397515873	p.Ile242Phe	26

CKD, chronic kidney disease; ESRD, end stage renal disease; HCM, hypertrophic cardiomyopathy; *3 cases reported EGL Genetic Diagnostics (Eurofins Clinical Diagnostics)

## Family A

37 year old male was referred to nephrology following the detection of skin lesions that were identified as angiokeratoma. He was noted to have reduced serum alpha-galactosidase levels. At the time his blood pressure and renal function were documented as normal (serum creatinine within reference range) with no evidence of proteinuria. Within 3 years his kidney function deteriorated and he unfortunately progressed to ESRD, requiring haemodialysis. He was also was noted to have a mild hypertrophic cardiomyopathy.

Separately, his older brother, aged 47 years, was also noted to have skin lesions, having previously had an ischaemic stroke at the age of 46. A diagnosis of Fabry disease was confirmed by reduced serum alpha galactosidase levels. He also was documented as having normal renal function (serum creatinine within reference range) and left ventricular hypertrophy (LVH). He was treated with ERT. Over the last decade there has been only a small decline in his renal function (eGFR 67 to 52 mL/min/1.73m ^2^) with no evidence of proteinuria.

Mutation analysis in the proband and his brother confirmed a pathogenic *GLA* mutation p.Thr412Serfs and cascade screening has allowed the offspring of the two brothers to be genetically tested. A daughter and a grandson were identified as inheriting this pathogenic allele but are currently asymptomatic. Interestingly, the brothers had a maternal aunt who was noted to have undergone renal transplantation for an unknown cause of ESRD. There was no other family history of renal disease.

## Family B

A 39 year old lady with no family history of Fabry disease presented to Renal Services with hypertension, proteinuria but preserved renal function. A renal biopsy demonstrated multiple myelin inclusion bodies, typical of Fabry disease. Genetic tests confirmed a diagnosis of Fabry disease( *GLA* c.999-1G>C) and allowed testing of her son and her sister, who were mutation negative. She commenced ERT approximately 1 year after presentation, and responded with a reduction in proteinuria. Her renal function has remained normal throughout (eGFR>90 mL/min/1.73m ^2^).

## Family C

**Figure 2.**
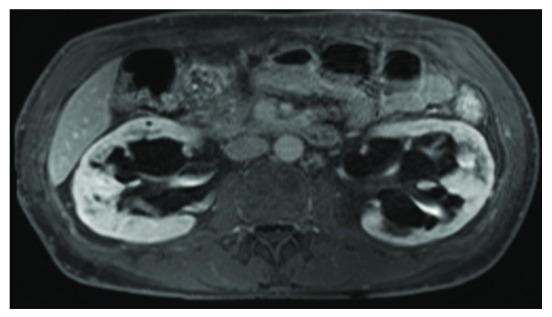
Parapelvic cysts in Fabry disease. MRI scan of proband from family C demonstrating bilateral parapelvic cysts.

A 38 year old man presented with proteinuria (Urine Protein Creatinine Ratio (UPCR) 56 µmol/mmol creatinine), preserved renal function (estimated Glomerular Fitration Rate (eGFR) 79 mL/min/1.73m ^2^) and a finding of possible renal cysts on ultrasound. He was also found to have a dilated left ventricle and aortic root on echocardiography. MR imaging of his kidneys demonstrated that his renal cysts were parapelvic in origin ( Figure 2), pointing to a diagnosis of Fabry disease. He underwent subsequent genetic testing which confirmed a *GLA* splicing mutation and commenced on ERT within a year of his presentation. He has no contactable family members. His renal impairment progressed despite treatment, reaching ESRD 10 years later at which point he received a pre-emptive live donor transplant.

## Family D

A 50 year old male was diagnosed with Fabry disease following presentation to neurology services with gait disturbance and thigh pain. Brain MRI revealed a previous ischaemic stroke. At the time of diagnosis he had evidence of CKD (eGFR 82 mL/min/1.73m ^2^) and proteinuria with macroscopically normal kidneys on ultrasound. He was also noted to have hypertrophic cardiomyopathy. He was commenced on enzyme replacement therapy. His renal function has declined since diagnosis 9 years ago and eGFR is now 43 mL/min/1.73m ^2^ with significant proteinuria (UPCR 108 µmol/mmol creatinine).

A year later his brother was referred to Renal Services aged 43 years with progressive renal dysfunction (estimated Glomerular Filtration Rate (eGFR) 57 mL/min/1.73m ^2^) and proteinuria. He had been made aware that his brother had a diagnosis of Fabry disease and his diagnosis was confirmed by low serum levels of alpha-galactosidase and genetic testing (missense mutation in *GLA* p.Ile117Ser). He was established on ERT but his renal function deteriorated over the last 10 years to an eGFR of 23 mL/min/1.73m ^2^. He is currently being worked up for a pre-emptive renal transplant. He is otherwise asymptomatic.

Cascade screening in this family revealed that some of the brothers’ relatives were also affected, including a male maternal cousin who had presented at 20 years of age with progressive CKD resulting in ESRD. His originally documented cause of ESRD was of unknown aetiology. He subsequently underwent genetic testing which confirmed the same GLA mutation (p.Ile117Ser) as his cousins.

This large family had a number of other relatives who have presented to Renal Services or have been diagnosed as a result of cascade screening, including a 32 year old male cousin (also on the maternal side) who presented late with ESRD. He had been made aware of the diagnosis of Fabry disease within the family and his diagnosis was confirmed on renal biopsy and by genetic testing. He is now established on haemodialysis and receives ERT. His 39 year old brother was also diagnosed with Fabry disease the following year and was commenced on ERT. He reported classic neurological symptoms of acroparathesia and gastrointestinal upset and had normal renal function and only microalbuminuria at presentation. Both brothers have young daughters who have mild neurological symptoms that may be attributed to Fabry disease however they have normal renal function and have not yet undergone genetic testing.

## Family E

A 59 year old man presented with severe hypertension that was refractory to anti-hypertensive therapy and he was referred to Renal Services for consideration of atypical causes. It came to light that his older brother had presented to Renal Services within the same centre 35 years before, aged 25 with severe hypertension and CKD. The older brother had rapidly progressed to ESRD within 3 years and was maintained on haemodialysis until he received a renal transplant aged 30 years. A review of the histological specimens taken at the time of a bilateral nephrectomy aged 34 (performed for severe hypertension) confirmed the presence of myelin inclusion bodies (zebra bodies) on electron microscopy.

A younger brother had undergone investigations over the years for a variety of symptoms and was also noted to have LVH, normal renal function but had documented microalbuminuria. The 3 brothers were found to have low levels of alpha-galactosidase activity and genetic analysis confirmed that all three siblings shared the identical p.Arg301Gln missense mutation. This degree of phenotypic variation within a family with the same genetic mutation is unusual and could potentially be explained by the role of environmental factors or additional modifying genes influencing disease manifestations ^27^. A number of relatives have been identified through cascade screening but are currently asymptomatic or have no evidence of renal impairment.

## Family F

A 45 year old female presented with proteinuria and progressive CKD but then developed cardiomyopathy. She had initially undergone a renal biopsy which reported findings consistent with focal segmental glomerular sclerosis (FSGS) however on review, the electron microscopy was found to have evidence of podocyte vacuolation in keeping with a diagnosis of Fabry disease. Mutation analysis confirmed a pathogenic *GLA* mutation p.Arg227*. A number of her relatives have been diagnosed with the same genetic mutation following cascade screening however they are currently asymptomatic with no evidence of renal involvement.

## Family G

A 23 year old male presented to renal services with haematuria, proteinuria and progressive CKD. He had classical features of Fabry disease including angiokeratoma and LVH. Genetic testing confirmed a p.Arg112Cys *GLA* mutation. Enzyme replacement therapy was commenced. Unfortunately, he had a progressive decline in renal function resulting in ESRD at the age of 33 years of age. He was treated with haemodialysis and renal transplantation. He was troubled with significant acroparasthesia. He died aged 41 years. Family screening confirmed that his mother and sister were carriers of the p.Arg112Cys variant. His mother lived to 77 years of age and died of congestive cardiac failure. His sister, aged 51 years has an absence of haematuria and proteinuria, with preserved renal function.

## Family H

A 32 year old male was referred to renal services with a finding of elevated serum creatinine and reduced eGFR (45mL/min/1.73m ^2^) and was also noted to have angiokeratoma. He underwent a renal biopsy which had electron microscopy findings consistent with a diagnosis of Fabry disease. He suffered with mild neuropathic pain but had no evidence of cardiac involvement. Mutational analysis of *GLA* revealed a nonsense mutation c.679C>T, p.Arg227*.

His brother was seen the following year aged 22 and was noted to have slightly reduced renal function (eGFR 79mL/min/1.73m ^2^) with no evidence of microalbuminuria but with evidence of left ventricular diastolic dysfunction on echocardiogram. Both brothers have been commenced on ERT and six other family members have been identified through cascade genetic screening.

## Discussion

Inherited renal disease is the fifth most common cause of ESRD ^28^. It is vital that a detailed family history of renal and extra-renal phenotypes is taken for any new patient presenting to renal services, whether the presentation is with hypertension, proteinuria or CKD. Only by doing this will the practitioner be able to piece together the often subtle clues that the patient may have an inherited disorder. If the family pedigree suggests or is compatible with X-linked inheritance (i.e. no evidence of male to male transmission) then Fabry disease should always be considered. Similarly the diagnosis should be considered in male patients with renal impairment and a finding of parapelvic cysts on renal ultrasound ^11^ as in the case of Family C. The European Best practice Group also recommend screening of any males under the age of 50 with unexplained CKD and to consider screening in females of any age ^19^.

Many of the manifestations of Fabry disease are non-specific and can occur in other systemic disorders such as diabetes and hypertension. As a result of this and a highly variable phenotype there is often a significant lag time between presentation to services and a diagnosis of Fabry disease. Females and atypically presenting males may only have hypertension or mild phenotypes and it may be difficult to recognize a pattern of inherited disease. Family D demonstrates how it may be helpful to revisit the family history of a patient with ESRD of unknown cause and consider Fabry disease as a potential diagnosis.

Renal biopsy, or as in the case of the patient in Family E, examination of renal tissue from nephrectomy, can often be helpful making the diagnosis. A small number of patients are identified co-incidentally through renal biopsy for investigation of CKD or proteinuria without any significant family history and when the diagnosis has not previously been considered. Screening for levels of alpha-galactosidase in at risk populations provides a non-invasive method of identifying patients. Similarly cascade genetic testing rapidly identifies affected family members in the absence of symptoms or clinical manifestations; potentially allowing treatment to be commenced earlier and prevention of disease progression.

## Conclusion

The pedigrees and disease spectrum we have described demonstrate the significant variation in phenotype, even amongst family members who have been confirmed to have the same genetic mutation. This along with variation in disease phenotype, including the existence of atypical variants of Fabry Disease, means that there is often a significant delay before patients receive a diagnosis and are therefore able to start ERT and other protective treatments. The importance of a high index of clinical suspicion of Fabry disease in patients with unexplained CKD and the determination of a full family history cannot be stressed enough. With the advent of both dry blood spot alpha-galactosidase testing and molecular genetic screening the speed and ability to detect affected family members has been significantly improved. This allows a precise diagnosis to be made and for patients to be commenced on ERT early in their disease course with the hope of preventing worsening of symptoms and organ damage.

## Consent

Written informed consent for publication of their clinical details and clinical images was obtained from the patients and/or relatives of the patients.

## Data availability

All data underlying the results are available as part of the article and no additional source data are required.

## References

[1] MacDermot KD Holmes A Miners AH: Anderson-Fabry disease: clinical manifestations and impact of disease in a cohort of 60 obligate carrier females. *J Med Genet.* 2001;38(11):769–75. 10.1136/jmg.38.11.769 11732485 PMC1734754

[2] Germain DP: Fabry disease. *Orphanet J Rare Dis.* 2010;5:30. 10.1186/1750-1172-5-30 21092187 PMC3009617

[3] Rigoldi M Concolino D Morrone A: Intrafamilial phenotypic variability in four families with Anderson-Fabry disease. *Clin Genet.* 2014;86(3):258–63. 10.1111/cge.12261 23980562

[4] Brennan P Parkes O: Case-finding in Fabry disease: experience from the North of England. *J Inherit Metab Dis.* 2014;37(1):103–7. 10.1007/s10545-013-9629-8 23828401

[5] West M Nicholls K Mehta A: Agalsidase alfa and kidney dysfunction in Fabry disease. *J Am Soc Nephrol.* 2009;20(5):1132–9. 10.1681/ASN.2008080870 19357250 PMC2678048

[6] Germain DP Charrow J Desnick RJ: Ten-year outcome of enzyme replacement therapy with agalsidase beta in patients with Fabry disease. *J Med Genet.* 2015;52(5):353–8. 10.1136/jmedgenet-2014-102797 25795794 PMC4413801

[7] Banikazemi M Bultas J Waldek S: Agalsidase-beta therapy for advanced Fabry disease: a randomized trial. *Ann Intern Med.* 2007;146(2):77–86. 10.7326/0003-4819-146-2-200701160-00148 17179052

[8] Ries M Gupta S Moore DF: Pediatric Fabry disease. *Pediatrics.* 2005;115(3):e344–55. 10.1542/peds.2004-1678 15713906

[9] Alroy J Sabnis S Kopp JB: Renal pathology in Fabry disease. *J Am Soc Nephrol.* 2002;13 Suppl 2:S134–8. 12068025

[10] Pisani A Petruzzelli Annicchiarico L Pellegrino A: Parapelvic cysts, a distinguishing feature of renal Fabry disease. *Nephrol Dial Transplant.* 2018;33(2):318–323. 10.1093/ndt/gfx009 28371803

[11] Sayer JA Haslam P Brennan P: Parapelvic cysts leading to a diagnosis of Fabry disease. *Kidney Int.* 2008;74(10):1366. 10.1038/ki.2008.141 18974770

[12] Ma TL Neild GH: Parapelvic cyst misdiagnosed as hydronephrosis. *Clin Kidney J.* 2013;6(2):238–9. 10.1093/ckj/sfs189 26019858 PMC4432443

[13] Branton M Schiffmann R Kopp JB: Natural history and treatment of renal involvement in Fabry disease. *J Am Soc Nephrol.* 2002;13 Suppl 2:S139–43. 12068026

[14] Schiffmann R Warnock DG Banikazemi M: Fabry disease: progression of nephropathy, and prevalence of cardiac and cerebrovascular events before enzyme replacement therapy. *Nephrol Dial Transplant.* 2009;24(7):2102–11. 10.1093/ndt/gfp031 19218538 PMC2698092

[15] Wanner C Oliveira JP Ortiz A: Prognostic indicators of renal disease progression in adults with Fabry disease: natural history data from the Fabry Registry. *Clin J Am Soc Nephrol.* 2010;5(12):2220–8. 10.2215/CJN.04340510 20813854 PMC2994083

[16] Germain DP Hughes DA Nicholls K: Treatment of Fabry's Disease with the Pharmacologic Chaperone Migalastat. *N Engl J Med.* 2016;375(6):545–55. 10.1056/NEJMoa1510198 27509102

[17] Hughes DA Nicholls K Shankar SP: Oral pharmacological chaperone migalastat compared with enzyme replacement therapy in Fabry disease: 18-month results from the randomised phase III ATTRACT study. *J Med Genet.* 2017;54(4):288–96. 10.1136/jmedgenet-2016-104178 27834756 PMC5502308

[18] Jain G Warnock DG: Blood pressure, proteinuria and nephropathy in Fabry disease. *Nephron Clin Pract.* 2011;118(1):c43–8. 10.1159/000320903 21071972

[19] Tsakiris D Simpson HK Jones EH: Report on management of renale failure in Europe, XXVI, 1995. Rare diseases in renal replacement therapy in the ERA-EDTA Registry. *Nephrol Dial Transplant.* 1996;11 Suppl 7:4–20. 9067983 10.1093/ndt/11.supp7.4

[20] Sessa A Meroni M Battini G: Chronic renal failure, dialysis, and renal transplantation in Anderson-Fabry disease. *Semin Nephrol.* 2004;24(5):532–6. 10.1016/j.semnephrol.2004.06.024 15490423

[21] Gal A: Molecular Genetics of Fabry Disease and Genotype–Phenotype Correlation.In: Elstein D, Altarescu G, Beck M, *Fabry Disease* Dordrecht: Springer Netherlands;2010;3–19. 10.1007/978-90-481-9033-1_1

[22] Shabbeer J Yasuda M Luca E: Fabry disease: 45 novel mutations in the alpha-galactosidase A gene causing the classical phenotype. *Mol Genet Metab.* 2002;76(1):23–30. 10.1016/S1096-7192(02)00012-4 12175777

[23] Ishii S Sakuraba H Suzuki Y: Point mutations in the upstream region of the alpha-galactosidase A gene exon 6 in an atypical variant of Fabry disease. *Hum Genet.* 1992;89(1):29–32. 10.1007/BF00207037 1315715

[24] Davies JP Winchester BG Malcolm S: Mutation analysis in patients with the typical form of Anderson-Fabry disease. *Hum Mol Genet.* 1993;2(7):1051–3. 10.1093/hmg/2.7.1051 8395937

[25] Yasuda M Shabbeer J Benson SD: Fabry disease: characterization of alpha-galactosidase A double mutations and the D313Y plasma enzyme pseudodeficiency allele. *Hum Mutat.* 2003;22(6):486–92. 10.1002/humu.10275 14635108

[26] Richfield L Bruce R Baker R: Phenotypical expression of mutation R227X exon 5 of the GLA gene (nucleotide change p.Arg227X c.608C > T) identified in a large UK kindred spanning eight generations in a well-documented family pedigree. *Acta Paediatr.* 2006;95:127–8. Reference Source

[27] Brady M Montgomery E Brennan P: Diagnosing Fabry disease--delays and difficulties within discordant siblings. *QJM.* 2015;108(7):585–90. 10.1093/qjmed/hct024 23378663

[28] Devuyst O Knoers NV Remuzzi G: Rare inherited kidney diseases: challenges, opportunities, and perspectives. *Lancet.* 2014;383(9931):1844–59. 10.1016/S0140-6736(14)60659-0 24856029 PMC4135047

