# Supplementary material for: Variable phenotypic presentations of renal involvement in Fabry disease: a case series
Source: F1000Res. 2018 Mar 22;7:356. [Version 1] doi: 10.12688/f1000research.13708.1 (PMC5930549; doi:10.12688/f1000research.13708.1)
Supplement: Peer review history

# Peer review: Referee response for version 1

**DOI:** 10.5256/f1000research.14890.r32323

**Published:** 2018-04-27

**Authors:** A. Neil Turner

**Affiliations:** 1 Renal Medicine, Royal Infirmary, University of Edinburgh, Edinburgh, UK

This well-written account from a renal unit gives a clear detailed account of the range of presentations of Fabry Disease in 8 local patients with mainly renal manifestations of this rare lysosomal storage disorder. An excellent summary of the condition is followed by details of index patients and relatives.

 The condition is increasingly recognised, but only some nephrologists are not fully aware of the range of presentations and manifestations. It is important as its very expensive treatments need careful consideration, though heavy proteinuria or other evidence of significant renal consequences would usually considered to be a treatment indication. The unexplained association with parapelvic cysts is nicely illustrated.

 There is no doubt that there are unrecognised patients still; whether they meet treatment criteria is important, and not addressed. But that's OK.

I have read this submission. I believe that I have an appropriate level of expertise to confirm that it is of an acceptable scientific standard.

# Peer review: Referee response for version 1

**DOI:** 10.5256/f1000research.14890.r32417

**Published:** 2018-04-26

**Authors:** Shabbir Moochhala

**Affiliations:** 1 UCL Centre for Nephrology, Royal Free Campus and Hospital, University College London Medical School, London, UK

This report describes families in whom a diagnosis of Fabry was made via renal services. This is a valuable piece of work and is a useful contribution to the renal literature. The descriptions are careful and succinct, and are complete with mutations analysis data and pedigree charts. Thus they illustrate the authors' main points which are of high clinical suspicion and attention to family histories in general renal clinics. There are also examples of rare presentations given, e.g. presentation with hypertension in a female patient and confirmed on biopsy.

 Some specific observations:

 Introduction: 

 Para 4: There is a differential diagnosis associated with zebra bodies. It may be worth noting this (e.g. antimalarials, amiodarone) as they can give false positives. 

 Para 8: Tahir, J Am Soc Nephrol 2007 Sep;18(9):2609-17 provides evidence that ACE/ARB use in addition to ERT is essential to reduce proteinuria, and this is regarded as standard practice i.e. it is more than "reasonable" as stated in para 8.

 Para 9: Ref 19 was an observational study performed in untreated Fabry disease (i.e. pre-ERT era) so this should be stated. 

 Cases:

 Family A, brother 2: Was he also treated for LVH? If so, an ACE or ARB may have augmented the response from ERT.

 Family E: Were other investigations performed to exclude other more common causes of hypertensive renal disease in the index patient? Such severe hypertension as an initial presentation of Fabry is regarded as very rare. Another diagnosis may have co-existed.

 Family F: It would be unusual to find no lamellated bodies anywhere on an initial electron microscopic report of a renal biopsy from a patient with Fabry.

 Discussion:

 Screening for alpha gal in at risk populations is only reliable in males, not the whole population.

I have read this submission. I believe that I have an appropriate level of expertise to confirm that it is of an acceptable scientific standard.
